# Cortical high-threshold and low-activation characteristics in adolescent depression: a cross-age differential analysis

**DOI:** 10.3389/fpsyt.2026.1800009

**Published:** 2026-05-14

**Authors:** Jialin Gai, Duanwei Wang, Fengya Zhen, Tao Kong, Xiuqing Niu, Xianwei Che, Shengqiao Wang, Zhe Liu, Cuixia An, Xu Lin

**Affiliations:** 1 Shandong Mental Health Center, Shandong University, Jinan, Shandong, China; 2 Department of Psychiatry, The First Hospital of Hebei Medical University, The Mental Health Center of Hebei Medical University, Shijiazhuang, Hebei, China; 3 The Affiliated Hospital of Hangzhou Normal University, Hangzhou, China

**Keywords:** functional Near-InfraRed Spectroscopy (FNIRS), Major Depressive Disorder (MDD), neuromodulation, resting motor threshold (RMT), repetitive transcranial magnetic stimulation (TMS)

## Abstract

**Background:**

Adolescent depression exhibits distinct neurophysiological features, with marked age heterogeneity particularly in the resting motor threshold (RMT) measured during repetitive transcranial magnetic stimulation (rTMS), and substantial variability in clinical therapeutic efficacy. Functional near-infrared spectroscopy (fNIRS) enables the assessment of cortical excitability levels; however, research investigating the association between RMT and cortical activation in adolescent patients with depression remains limited. This study aims to elucidate the underlying neural mechanisms from the perspective of cortical hemodynamics, which is crucial for further optimizing neuromodulation strategies in patients with depression.

**Methods:**

We collected data from 85 treatment-naive patients with depression who underwent rTMS therapy. All patients completed RMT measurement, fNIRS examination, and Hamilton Depression Rating Scale (HAMD) assessment prior to rTMS treatment. Participants were divided into three groups according to age: the adolescents group (n=31), the young adult group (n=26), and the middle-aged group (n=28). We compared the differences in RMT among the three groups and explored the relationships between RMT, cortical activation (reflected by prefrontal oxyhemoglobin level changes during the verbal fluency task via fNIRS), and depression severity (assessed by HAMD scores).

**Results:**

The results demonstrated that the adolescents group had a significantly higher RMT than the other age groups (58.00 ± 11.14, *P* < 0.001), accompanied by the lowest prefrontal Oxy-Hb activation level (0.095 ± 0.06, *P* < 0.001). A strong negative correlation was observed between RMT and cortical activation (Spearman’s rs= -0.929, *P* < 0.001), while a strong positive correlation was found between RMT and depression severity (*Spearman’s rs* = 0.837, *P* < 0.001). The distinct coupling phenomenon of high threshold-low activation-severe symptoms was most prominently manifested in this age group, which may theoretically reflect an underlying dysregulation in broader emotional networks, though the current direct findings strictly indicate localized alterations in prefrontal activation and motor cortical excitability.

**Conclusions:**

The characteristics of high RMT and low cortical activation in adolescent depression serve as important neurobiological markers for depression severity. This finding provides a novel direction for developing individualized, developmentally tailored neuromodulation strategies (e.g., optimization of rTMS targets and dosages), indicating that interventions for adolescent depression should prioritize promoting the healthy integration of emotional circuits and the functional coordination between the cortex and subcortex.

## 1 Introduction

Globally, adolescent depression has emerged as an imminent public health crisis. Beyond its high prevalence and disability burden, the disorder derails critical developmental trajectories in cognition, emotion and social integration underscoring the urgency of elucidating its pathological mechanisms and developing tailored intervention strategies (1). While traditional diagnosis and assessment hinge on subjective symptom descriptions; however, the adolescent brain resides in a unique developmental flux of dramatic structural and functional remodeling. Consequently, the neurobiological underpinnings of depression in this population may diverge significantly from adults models (2). Therefore, shifting the research focus from behavioral phenotypes to the underlying neural circuits and physiological bases is essential for understanding the unique nature of adolescent depression.

To date, neuroimaging studies have begun to delineated the neural circuit models of adolescent depression, with core abnormalities frequently involving dysfunctional connectivity within the prefrontal-limbic system circuit which is responsible for cognitive control and emotional regulation (3). Some abnormalities in high-order circuits may originate from the disruption of the more fundamental balance between cortical excitation and inhibition. Transcranial magnetic stimulation (TMS), a non-invasive tool with both therapeutic and diagnostic capabilities, provides a means to directly test this hypothesis. In rTMS therapy, the precise setting of stimulation parameters is one of the key factors ensuring therapeutic efficacy, including stimulation intensity, frequency, target site, and treatment course (4, 5). Among these parameters, stimulation intensity is typically calibrated based on the resting motor threshold (RMT), defined as the minimum stimulation intensity capable of eliciting a minimal observable contraction in a target muscle (e.g., the first dorsal interosseous muscle of the hand) under a relaxed state (6). RMT is generally regarded as a measure of the membrane excitability of corticospinal neurons and interneurons in the motor cortex, reflecting the baseline level of individual cortical excitability. It plays a crucial role in ensuring the safety and effectiveness of treatment and has become an important window for exploring the neurophysiological mechanisms of depression (7, 8). Importantly, adolescence is characterized by profound neurodevelopmental changes, including hormonal fluctuations, ongoing myelination, and dynamic remodeling of cortical E–I balance, all of which may influence neural plasticity and responsiveness to stimulation. Directly extrapolating RMT percentages from adults to children may result in excessively strong or weak stimulation; thus, understanding the age-specific distribution of RMT is critical for establishing safe and effective dosage regimens.

Existing research has suggested age-related differences in cortical excitability and clinical symptoms among patients with depression; however, empirical studies specifically targeting adolescents and directly comparing resting motor threshold (RMT) across age groups remain limited (9). Neurophysiological evidence indicates that the excitation-inhibition balance in adolescents is highly dynamic and susceptible to excessive activation of emotional networks, with structural and functional imaging findings showing partial similarities to adults but exhibiting distinct developmental specificity (10). A research team from the University of Helsinki Hospital in Finland provided direct evidence for such age-related differences by evaluating the cortical regulatory characteristics of spinal cord excitability in children and adolescents (11). This study found that, unlike adults, children exhibited significant early facilitation at a stimulation intensity of 90% RMT, suggesting that developing spinal motor neurons are more sensitive to cortical inputs. Although this study did not focus on depressive populations, it offers important neurophysiological insight into developmental differences in excitability thresholds that may contribute to age-related variability in RMT. Concurrently, a cohort study revealed that most adult psychiatric disorders should be redefined as extensions of adolescent-onset conditions; patients with depression that onset in childhood or adolescence have a higher risk of recurrence in adulthood and also display lower cortical excitability (12). However, some researchers hold contrasting views: in elderly patients, structural and functional changes in the brain, microangiopathy, and cortical atrophy may impair the penetration and conduction efficiency of rTMS electromagnetic fields (13). These structural alterations may necessitate adjustments to stimulation parameters in elderly patients, such as appropriate increases in stimulation intensity or modifications to coil position, to compensate for the effects of brain structural changes. In contrast, the adolescent brain is in a developmental stage, with neuronal excitability and plasticity differing from those of adults. Therefore, more moderate parameters and age-specific stimulation intensities may be required during treatment to ensure safety and achieve optimal therapeutic outcomes (14, 15). These age-related differences in RMT not only have practical clinical implications but may also reflect underlying differences in the neurophysiological mechanisms of depression across age groups. Most current rTMS studies adopt relatively uniform protocols, potentially overlooking age-related neurophysiological differences. As clinical symptoms improve in patients with depression, cortical excitability undergoes corresponding changes, and the underlying neurophysiological mechanisms exhibit significant age heterogeneity (16). However, how these differences influence treatment outcomes and their relationship with depression severity remain unclear. Furthermore, few studies have integrated functional near-infrared spectroscopy (fNIRS) into this field to quantify cortical activation levels from a hemodynamic perspective and elucidate the interactive relationships between cortical activation, RMT, and depression severity. With its advantages of high tolerability and resistance to motion artifacts, fNIRS has emerged as an ideal tool for probing prefrontal cortical activation states in adolescents (17, 18). By combining TMS-RMT with fNIRS, we can construct a physiology-function correlation framework that explores the baseline excitability of various brain regions and how it couples with the neural activation efficiency of the dorsolateral prefrontal cortex (DLPFC) during task performance. This multimodal research strategy holds promise for bridging local physiological indicators with the global function of emotional regulatory circuits, thereby providing a more integrated perspective for understanding adolescent depression.

In conclusion, age plays a pivotal role in the neurophysiological underpinnings of depression. Based on the unique characteristics of adolescent depression and the core context of neural development, we hypothesize that age-related heterogeneity in RMT is directly associated with cortico-subcortical interactions and clinical symptoms in this population. Successful cross-age comparisons in this regard will have direct clinical application value for the future rTMS treatment of major depressive disorder (MDD). Therefore, understanding the age-related differences in cortical excitability (measured via RMT) and prefrontal activation (assessed via fNIRS), and how these parameters jointly characterize adolescent depression, is necessary for advancing personalized rTMS interventions. This study aims to reveal the unique cortical physiological characteristics of adolescent depression through cross-age differential analysis combined with fNIRS, providing critical evidence for the subsequent optimization of individualized assessment and age-targeted neuromodulation strategies in patients with depression.

## 2 Materials and methods

### 2.1 Participants

The data used in this study were obtained from the Neuroregulation Center of Shandong Mental Health Center. All patients signed written informed consent forms to receive rTMS treatment. This study was approved by the Ethics Committee of Shandong Mental Health Center on December 4, 2025 (No. of ethical approval:KYSJWLL2025-1-089).

Inpatients who voluntarily received rTMS treatment from June 2024 to June 2025 were collected. The inclusion criteria were:(a) meeting the diagnostic criteria for major depressive disorder in the diagnostic and statistical manual of mental disorders,5th Edition(DSM-5) (19);(b) the age of the subjects ranges from 12 to 59 years old.(c)first-visit time Patients with Hamilton Depression Scale-24(HAMD-24) scores of 20-35;(d)the subjects completed the nerve conduction velocity and motion threshold detection as well as the near-infrared brain functional imaging detection conducted by our center.(e) normal vision and hearing, right-handed. Exclusion criteria were:(a) participants with cochlear implants, pacemakers or other metal implants;(b) severe systemic diseases, including cardiovascular or cerebrovascular diseases, hepatic or renal dysfunction, infections, malignant tumors, or autoimmune disorders;(c) history of neurological diseases such as head trauma, epilepsy, and dementia; (d) presence of any psychiatric comorbidities, such as bipolar disorder, schizophrenia spectrum disorders, or primary anxiety disorders; (e) use of central nervous system–affecting medications, illicit drugs, or alcohol within three days prior to testing;(f) poor compliance or inability to complete RMT or fNIRS assessments.

Patients with HAMD-24 scores exceeding 35 were excluded from the study. This restriction was implemented because extremely severe depressive symptoms are frequently accompanied by significant psychomotor retardation, which hinders the patients’ ability to effectively cooperate and complete the VFT during fNIRS recording, thereby confounding the hemodynamic data. Additionally, such patients often require immediate acute interventions due to higher clinical risks. The 3-day restriction for alcohol and CNS-affecting medications was adopted to minimize their acute transient effects on cortical excitability and hemodynamic responses. Furthermore, because this study exclusively enrolled treatment-naive patients, none of the participants had a history of habitual psychotropic medication use. Individuals with a DSM-5 diagnosis of substance or alcohol use disorders were strictly excluded during the clinical screening phase.

After screening, a total of 85 patients with depression met the criteria. Participants were classified into three age cohorts: adolescents group (12–18 years,n=31), young adults group (19–39 years,n=26), and the middle-aged adults group (40–59 years,n=28).

### 2.2 Clinical assessment

Collect demographic information of the participants. In addition to demographic information, the participants’ disease course history, family history of mental illness, suicide situations, and conditions of concomitant physical diseases were also collected. After admission, participants were evaluated for the severity of depression by their psychiatrists or psychotherapists using the HAMD-24 scale. The 24-item HAMD includes 24 items rated on either a 2-, 3- or 4- point scale with total score range from 0 to 76 points (20).Patients with a total score of 8 to 19 in HMAD-24 are regarded as having mild depression, those with a total score of 20 to 35 are regarded as having moderate depression, those with a total score of >35 are regarded as having severe depression, and those with a total score of less than 8 have no depressive symptoms. Participants included in this study need to score between 20 and 35 points.

### 2.3 Neurophysiological measurement

#### 2.3.1 Overall experimental procedure

Following admission and initial clinical evaluation (including HAMD-24 scoring), all eligible patients underwent a standardized neurophysiological assessment protocol in a quiet testing environment prior to receiving any rTMS treatment. The procedure was conducted sequentially. First, the baseline RMT was determined using single-pulse TMS over the left primary motor cortex to gauge inherent cortical excitability. Immediately thereafter, participants were fitted with the fNIRS optode cap. Once optimal optode-to-scalp contact and signal calibration were confirmed, subjects performed a structured VFT paradigm lasting 150 seconds. Driven by automated voice prompts, this paradigm consisted of three phases: an initial 30-second preparation and baseline period (counting backward or resting with eyes closed), a 60-second active task phase (generating words based on four specific semantic cues, 15 seconds per cue), and a final 60-second recovery phase to allow cerebral hemodynamics to return to baseline. Continuous Oxy-Hb variations were recorded throughout this entire sequence.

#### 2.3.2 RMT measurement

All enrolled subjects underwent RMT testing using a TMS device (Model: MagNeuro X180, Nanjing Vishee Medical Technology Co., Ltd., Nanjing, China) after admission to the hospital and before receiving rTMS treatment. In a comfortable and quiet assessment room, subjects were instructed to sit on a high-back chair with armrests, keeping the muscles of both upper limbs relaxed, and placing the right forearm on the armrest with the palm facing upward in a natural manner. The assessor then had the subjects wear a positioning cap. In accordance with the international 10–20 electroencephalography (EEG) positioning standard (21), the hand representation area in the primary motor cortex was located using C3 as the reference point. Subjects were also reminded not to move their heads arbitrarily during the test to ensure the TMS coil remained in the same position leads were connected next. The recording electrodes were attached to the muscle belly of the right abductor pollicis brevis, the reference electrodes to the tendon of the right APB, and the ground electrode to the wrist. The skin at the electrode attachment sites was wiped with an alcohol wipe to ensure sufficiently low skin impedance.

A figure-of-eight coil was used with the device to deliver single-pulse stimulation to the subject’s left motor cortex. The device could automatically adjust the coil position to locate the hand area of the primary M1 in the subject. When the “hot spot” (optimal stimulation site) was found, the device would automatically adjust the stimulation intensity. By observing the motor evoked potentials collected in the software, when the intensity approached the threshold, the output intensity of the device would gradually decrease. This process continued until the minimum output intensity could elicit at least 5 MEPs with an amplitude greater than 50 μV out of 10 consecutive TMS stimulations. This minimum output intensity was defined as the RMT. Any MEPs affected by other muscle activities or with an amplitude less than 50 μV were excluded from the analysis (22).

#### 2.3.3 fNIRS recording and preprocessing

In this study, fNIRS data were acquired using a near-infrared brain function imaging system (Model: BS-7000,Wuhan Yiruide Group,Wuhan,China). Near-infrared light with two wavelengths (690 nm and 830 nm) was used to measure the concentration changes of oxygenated hemoglobin (Oxy-Hb) and deoxygenated hemoglobin (Deoxy-Hb) within 2–3 cm below the cerebral cortex. The system consists of 24 light sources (emitters) and 24 light detectors, with a distance of 3 cm between each emitter and detector, forming a total of 53 measurement channels. The correspondence between the NIRS channels and the measurement positions on the cerebral cortex was confirmed, and positioning was performed in accordance with the international 10–20 electroencephalography electrode system. Specifically:

Prefrontal cortex channels: 16, 17, 19–23, 27–31, 33–37, 41.
Left temporal lobe channels: 2, 3, 5–9, 13–15.
Right temporal lobe channels: 42–46, 48–51, 53.

**Figure 1:**
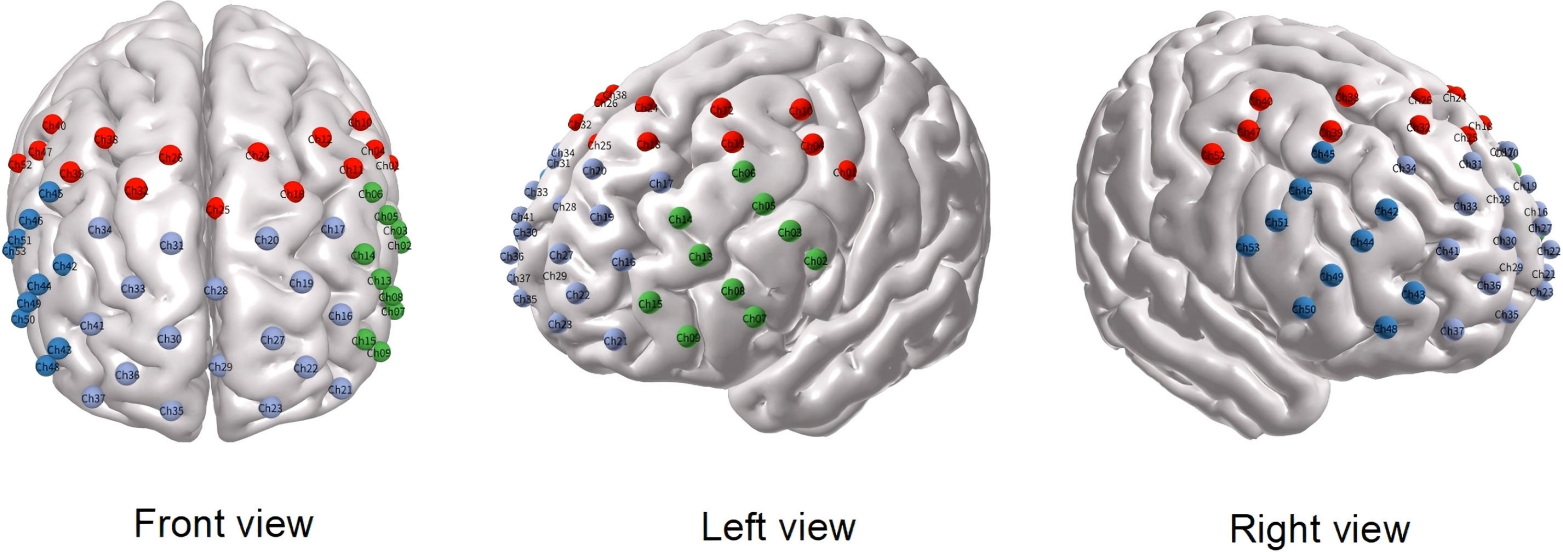
54 Location of probes and channel settings in 54-channel near-infrared spectroscopy. Brodmann area showing 54 sensing areas (from Ch1 to Ch54) within the prefrontal cortex 54.Purple represents the prefrontal channel area; red represents the parietal channel area; green represents the left temporal channel area; blue represents the right temporal channel area.

The blood oxygen concentration of one channel could be measured between each pair of optodes (light emitter and detector). The channel distribution map is shown in **Figure 1**. Prior to data collection, the fNIRS cap was fitted to each participant, ensuring optimal optode-to-scalp contact. During the measurement, the surrounding environment was kept quiet, with no noise or unrelated personnel present. First, the subject’s basic information was entered, followed by a calibration phase to ensure subject comfort and optimal signal quality across all channels. After debugging, the formal testing phase began. Subjects were instructed to complete a standardized block-design VFT guided by automated audio prompts. The 150-second paradigm consisted of three continuous phases: a 30-second pre-task baseline (during which subjects counted numbers continuously to stabilize baseline signals), a 60-second active task phase, and a 60-second post-task recovery phase (resuming counting). During the active task phase (30s–90s), subjects were auditorily presented with four common Chinese characters (e.g., flower, home), with 15 seconds allocated to each character. Subjects were required to overtly generate as many legitimate words as possible containing the target character. (Note: Because the primary objective of this clinical assessment was to capture task-evoked prefrontal hemodynamic responses, precise behavioral performance metrics, such as total word counts, were not quantitatively recorded or analyzed). During the VFT, the concentrations of Oxy-Hb and Deoxy-Hb in the prefrontal region and bilateral temporal regions were continuously measured. After the test, the integral value, centroid value, and slope value of each brain region were calculated, with the following definitions: Integral value: Represents the change in Oxy-Hb concentration during the VFT, reflecting the intensity of the hemodynamic response. Centroid value: Represents the time required for the hemodynamic response intensity to reach half of its maximum; Slope value: Represents the spectral activation speed within the first 5 seconds after the start of the VFT;Information calibration was performed before each test to ensure the valid acquisition of fNIRS data.

The fNIRS data were preprocessed using NirMaster software (Wuhan Yiruide Group, China), which employs an automated and standardized pipeline commonly used in clinical settings (23). The specific steps and their methodological justifications are as follows: (1) Resampling to 10 Hz: This step was implemented to reduce data redundancy while adequately capturing the slow temporal dynamics of hemodynamic responses. (2) Conversion of optical intensity to optical density. (3) Motion artifact correction via spline interpolation: This algorithm was applied to identify and correct sudden signal jumps caused by head movements, thereby improving the signal-to-noise ratio (24). (4) Band-pass filtering (0.01–0.5 Hz): This filter was utilized to remove high-frequency physiological noise (e.g., cardiac pulsations and respiration) and low-frequency instrument drifts. (5) Application of the modified Beer-Lambert law: This is the established standard method used to convert optical density into concentration changes of Oxy-Hb and Deoxy-Hb (23). Although both chromophores were recorded by the system, this study focused exclusively on Oxy-Hb concentrations to quantify prefrontal activation. This analytical decision was based on two primary factors. First, Oxy-Hb typically presents a higher signal-to-noise ratio and demonstrates greater sensitivity to task-induced hemodynamic variations (neurovascular coupling) compared with Deoxy-Hb. Second, utilizing Oxy-Hb as the principal metric aligns our methodology with the predominant consensus in recent psychiatric fNIRS literature, thereby facilitating direct cross-study comparisons of cortical excitability in depressive disorders. The intensity of the Oxy-Hb signal is positively correlated with the degree of brain activation—specifically, a larger parameter estimate indicates more significant activation of the brain region.

### 2.4 Statistical analysis

Statistical analyses were performed using SPSS 26.0 software. Normality was assessed using the Shapiro-Wilk test. Continuous variables were assessed for normality using the Shapiro-Wilk test. Because several key variables (e.g., age, illness duration, Oxy-Hb changes, HAMD scores, and RMT) significantly deviated from a normal distribution, they were presented as the median and interquartile range (IQR). Consequently, non-parametric Spearman’s rank correlation analysis was utilized for all correlational assessments. Categorical data were presented as frequencies and percentages (%). Homogeneity of variance was also evaluated prior to group comparisons. To compare the RMT across the three groups, Type II sum-of-squares analysis of covariance (ANCOVA) was applied to compare RMT across the three groups while controlling for specified clinical covariates (gender, suicidal status, and illness duration). Subsequently, Spearman’s rank correlation analysis was used to explore the relationships among RMT, prefrontal cortical Oxy-Hb activation levels, and HAMD scores. Given the heterogeneity of variance, Welch’s analysis of variance (ANOVA) was employed to compare Oxy-Hb activation levels among the three age groups, followed by Bonferroni-corrected *post-hoc* pairwise comparisons. For the adolescent group, a multiple linear regression model was established to evaluate the independent effects of Oxy-Hb changes and HAMD scores on RMT, after adjusting for covariates including gender, disease duration, suicidal behavior, and family history of mental disorders. In all analyses, a two-tailed P value < 0.05 was defined as statistically significant. Additionally, a *post-hoc* power analysis was conducted using G*Power* software (version 3.1.9.7) to ascertain the statistical adequacy of the sample size. Based on the observed effect sizes for the primary analyses (ANCOVA for cross-age RMT comparisons and multiple linear regression in the adolescent group), the achieved statistical power (1-β) exceeded 0.95 at an alpha level of 0.05, confirming sufficient statistical power to detect the reported effects.

## 3 Results

### 3.1 Demographic and clinical characteristics

As shown in **Table 1**, there were no significant differences among the three groups in terms of gender (*P* = 0.087), family history of mental disorders (*P* = 0.404), comorbid physical illnesses (*P* = 0.637), suicidal behavior (*P* = 0.547), and HAMD scores (*P* = 0.413). By contrast, significant age differences were observed across the three groups: the median age of the adolescent group was 16.00 (IQR: 15.00 - 17.00) years, that of the young adult group was 27.85 ± 6.73 years, and that of the middle-aged group was 53.07 ± 4.17 years. The mean disease duration of patients in the middle-aged group (38.29 ± 31.40 months) was longer than that of patients in the adolescents and young adult groups.

**Table 1:** Demographic and clinical characteristics of participants.

Characteristics	Adolescent group (n=27)	Young adult group (n=26)	Middle-aged group (n=28)
Age (years)	16.00 (15.00 - 17.00)	27.00 (21.25 - 33.75)	53.00 (49.00 - 56.25)
Course of disease (months)	6.00 (4.50 - 12.00)	11.00 (6.00 - 23.25)	32.50 (12.00 - 50.00)
HAMD score	22.00 (21.00 - 23.50)	21.00 (20.00 - 23.00)	21.00 (20.00 - 23.25)
Oxy-Hb change	0.11 (0.06 - 0.12)	0.15 (0.13 - 0.22)	0.99 (0.57 - 1.17)
RMT (% MSO)	52.00 (48.50 - 65.00)	38.00 (34.00 - 44.50)	38.50 (35.00 - 47.50)

Data are presented as mean ± standard deviation (SD) for continuous variables and as number (percentage) for categorical variables. The numbers in parentheses represent the percentages within each respective group.

### 3.2 Analysis of covariance for resting motor threshold among the three groups

After adjusting for the effects of covariates, there was an extremely significant difference in RMT across the three groups (*F* = 44.5575, *P* < 0.001). As shown in **Table 2**, three potential confounding covariates were controlled during the analysis; whereas gender (*F* = 1.33, *P* = 0.253), suicidal status (*F* = 0.01, *P* = 0.909), and disease duration (*F* = 0.04, *P* = 0.841) had no significant impact on RMT results. Pairwise comparisons revealed that the RMT of the adolescent group was significantly higher than that of the young adult group ( P < 0.001, Cohen’s d = 1.571, 95% CI: [12.61,24.78]) and the middle-aged group ( t = 4.064, P < 0.001, Cohen’s d = 1.060, 95% CI: [7.17,20.90]). The young adult group had the lowest RMT ( 39.31 ±9.61), with no significant difference observed when compared to the middle-aged group ( t = −1.606, P = 0.114, Cohen’s d = −0.437, 95% CI: [–10.40,1.09]).

**Table 2:** Key results of analysis of covariance (ANCOVA) for RMT thresholds among three patient groups.

Category	Subcategory	Adolescent group (n=27)	Young adult group (n=26)	Middle-aged group (n=28)	Statistical outcomes
RMT Threshold	Original (Median (IQR))	52.00 (48.50 - 65.00)	38.00 (34.00 - 44.50)	38.50 (35.00 - 47.50)	–
	Adjusted¹ (EMM ± SE)	58.00 ± 11.14	39.31 ± 9.61	43.96 ± 10.57	–
ANCOVA Main Effect²	F-value	–	–	–	44.56
	P-value	–	–	–	<0.001***
Covariate Effects	Covariate	F-value	P-value	Significance Mark	–
	Gender	1.33	0.253	–	–
	Suicidal Status	0.01	0.909	–	–
	Illness Duration	0.04	0.841	–	–

This table integrates the core findings of Analysis of Covariance (ANCOVA) for Resting Motor Threshold (RMT) comparisons across three patient groups (adolescent, young adult, and middle-aged). Original RMT values are presented as median (interquartile range, IQR) due to their non-normal distribution. ¹Adjusted RMT: Expressed as Estimated Marginal Means ± Standard Error (EMM ± SE) after controlling for three potential confounders: gender, suicidal status, and illness duration (months). ²*Post-hoc* details: Adolescent vs. Young Adult (mean difference = 18.69, P<0.001, 95% CI: [12.28, 25.10]); Adolescent vs. Middle-aged (mean difference = 14.04, P<0.001, 95% CI: [7.82, 20.26]); Young Adult vs. Middle-aged (mean difference = -4.66, P=0.297, 95% CI: [-11.07, 1.76]). ³Significance: *** P<0.001 (statistical significance level: α=0.05); no mark indicates P≥0.05.

### 3.3 Relationships between resting motor threshold, cortical activation level, and depression severity

Finally, the mean concentration changes of Oxy-Hb in the prefrontal cortex (Channels 16, 17, 19–23, 27–31, 33–37, 41) were selected as the primary outcome measure for analysis.

**Figure 2:**
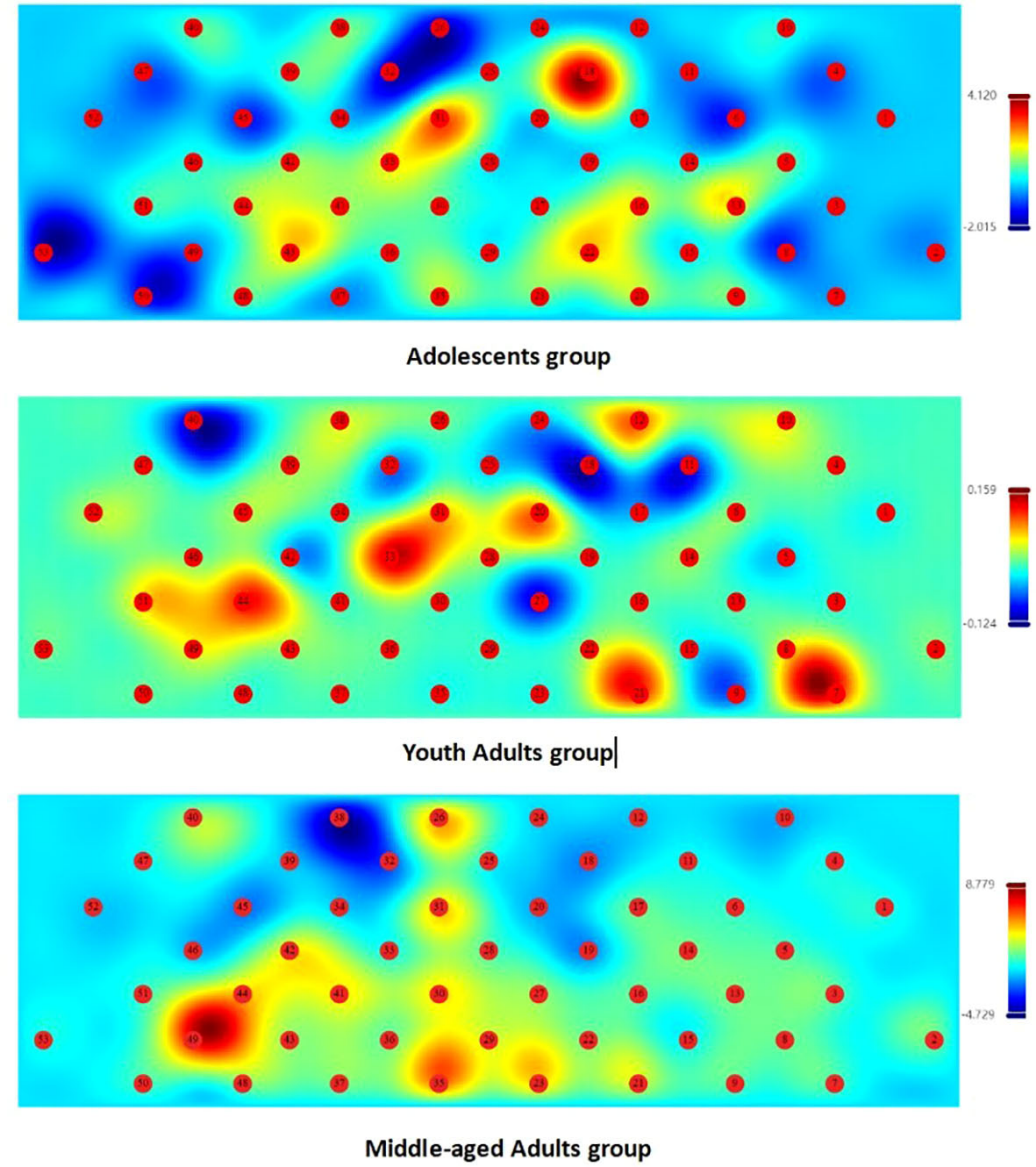
The concentration changes in Oxy-Hb activation levels across all channels in the three patient groups during the VFT. The legend on the right uses a blue-to-red color gradient, where the color transitions from blue (representing the lowest activation level) to red (representing the highest activation level). The color contrast represents the mean Oxy-Hb concentration difference between the active verbal fluency task phase and the baseline period. Note: These topographies are descriptive visual representations generated automatically by the NirMaster clinical software without channel-wise statistical thresholding (e.g., multiple comparison corrections).

Comparative analysis revealed that during the VFT, the adolescents group exhibited the lowest Oxy-Hb activation level (0.095 ± 0.06) among the three groups, particularly in the rDLPFC. The young adult group showed an intermediate activation level (0.162 ± 0.08), while the middle-aged group had the highest activation level (1.048 ± 0.58), with the most prominent activation also observed in the rDLPFC region. Significant differences in Oxy-Hb change levels were observed among the three age groups (Welch’s F{(2, 29.73)} = 203*2.47*, *P* < 0.001). *Post hoc* analysis revealed that the Middle-Aged group had significantly higher Oxy-Hb change levels than both the Young Adults group (*P* < 0.001) and Adolescents group (*P* < 0.001). The Young Adults group also showed significantly higher levels compared to the Adolescents group (*t* = -3.5218, *P* = 0.003).All pairwise comparisons demonstrate extremely significant differences (*P* < 0.001). Changes in activation levels across all channels during the task period in the three groups are illustrated in **Figure 2**.

**Figure 3:**
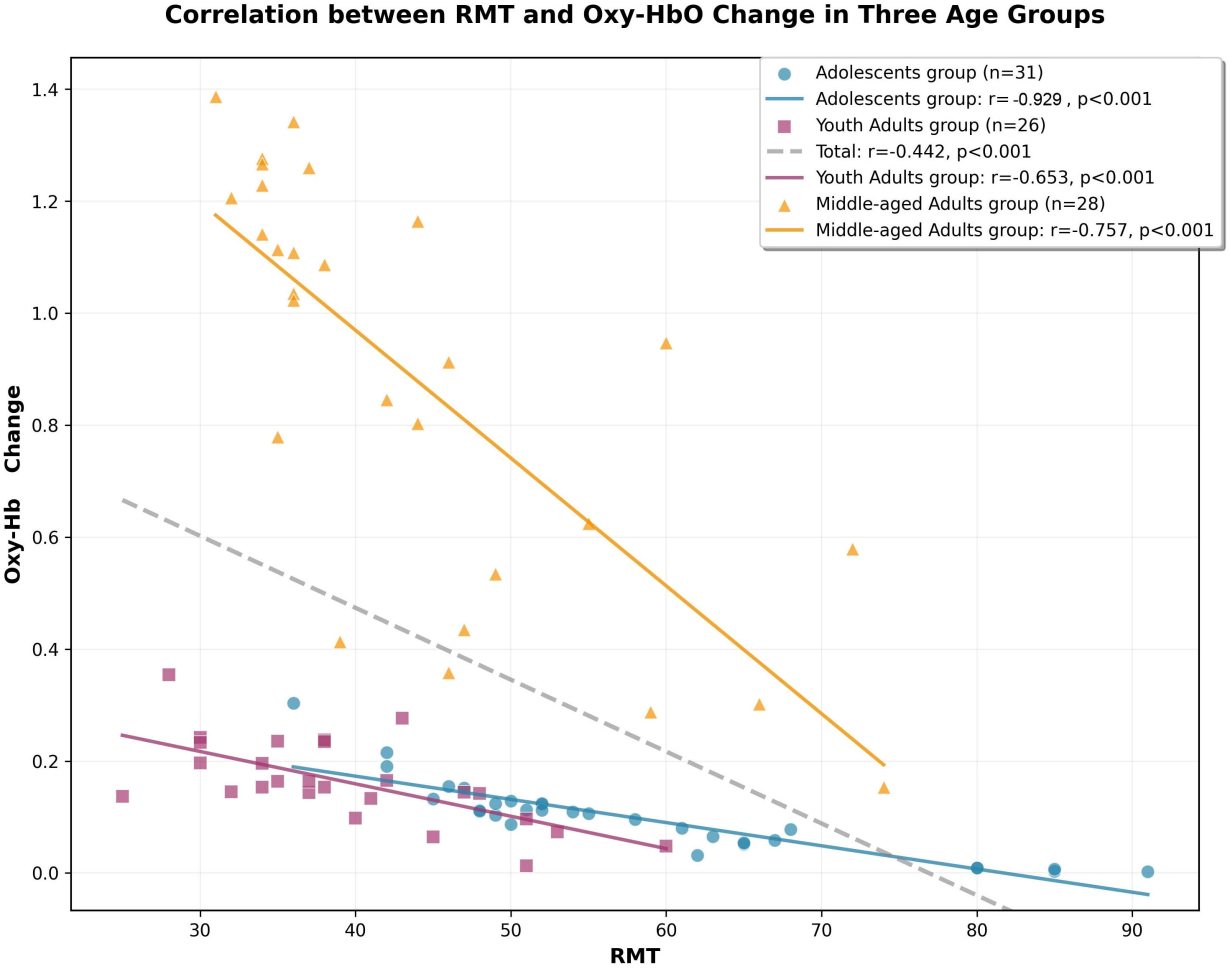
Correlation scatter plot between RMT and changes in[oxy-Hb] value in Three Age Groups. A scatter plot illustrating the relationship between RMT and Oxy-Hb Change across three age groups of patients. Each data point represents an individual patient, with different colors and markers distinguishing the groups: blue circles (○) for the Adolescents group (n=31), red squares (□) for the Youth Adults group (n=26), and orange triangles (△) for the Middle-aged Adults group (n=28). Solid colored lines represent the linear regression fit for each respective group, while the dashed gray line represents the linear regression fit for the total sample (n=85).

Subsequently, to investigate the association between transcranial magnetic stimulation-resting motor threshold (TMS-RMT) and cerebral activation intensity, we performed a correlation analysis between RMT data and the corresponding mean Oxy-Hb concentrations in the prefrontal cortex across the three groups. The results revealed a strong negative correlation between RMT scores and prefrontal activation in the total sample (*Spearman’s rs* = -0.442, *P* < 0.001). Notably, while significant negative correlations were observed across all cohorts—including the young adult (*Spearman’s rs* = -0.653, *P* < 0.001) and middle-aged groups (*Spearman’s rs* = -0.757, *P* < 0.001)—the association was most pronounced in the adolescents group (*Spearman’s rs* = -0.929, *P* < 0.001), These findings are presented in **Figure 3**.

**Figure 4:**
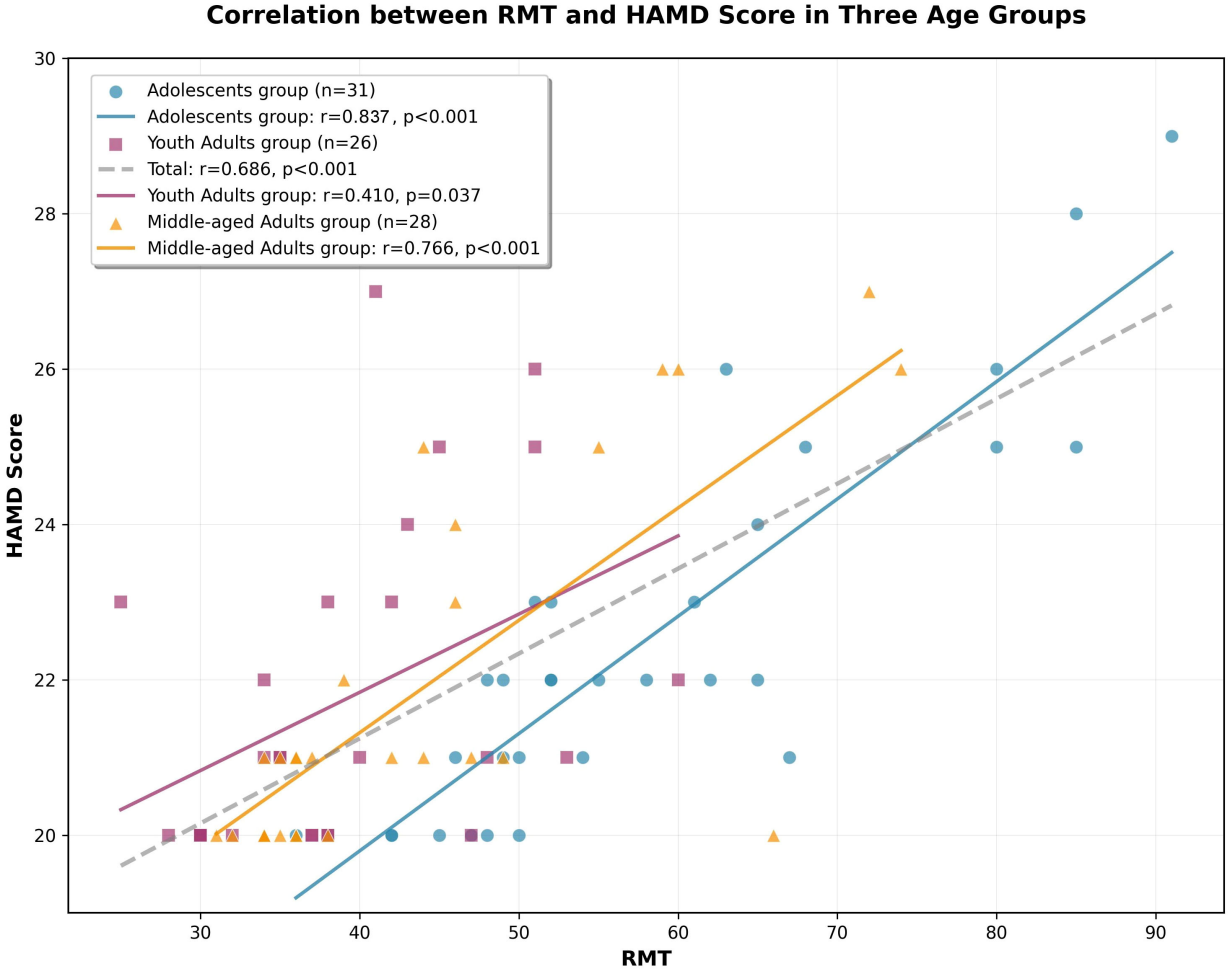
Correlation scatter plot between HAMD score and RMT in Three Age Groups.

To investigate the relationship between RMT and depression severity in patients, a correlation analysis was conducted between RMT and HAMD scores across the three groups. A strong positive correlation was observed between RMT and HAMD scores in the total sample (*Spearman’s rs* = 0.686, *P* < 0.001), As illustrated in **Figure 4**, the strongest correlation was identified in the adolescents group (*Spearman’s rs* = 0.837, *P* < 0.001), Parallel analyses in the other cohorts demonstrated relatively weaker yet statistically significant positive correlations for both the young adult (*Spearman’s rs* = 0.410, *P* = 0.037) and middle-aged groups (*Spearman’s rs* = 0.766, *P* < 0.001), which might be attributed to compounding confounding factors (e.g., medical comorbidities or chronic illness duration) prevalent in these older demographics.

### 3.4 Multiple linear regression analysis for the adolescents group

The aforementioned results indicated that the adolescents group exhibited the strongest correlation strength in both correlation analyses, suggesting that age may be a crucial factor influencing these associations. Therefore, we further performed a multiple linear regression model analysis specifically for the adolescents group. After controlling for potential confounding factors including gender, disease duration, suicidal behavior, and family history of mental disorders, the associations between the two primary independent variables and RMT remained statistically significant (*P* < 0.001). As shown in **Table 3**, none of the clinical covariates exerted a statistically significant effect on RMT, including gender (*t* = 0.87, *P* = 0.392, 95% CI: [-2.24, 5.52]), disease duration (*t* = -0.91, *P* = 0.370, 95% CI: [-0.44, 0.17]), suicidal behavior (*t* = -0.89, *P* = 0.383, 95% CI: [-6.01, 2.39]), and family history of mental disorders (*t* = 1.00, *P* = 0.327, 95% CI: [-2.21, 6.36]).

**Table 3:** Multiple linear regression analysis of RMT and related variables in the adolescent group.

Variable	Regression coefficient (β)	SE	t-value	P-value	95% CI	Adjusted R^2^
Constant	9.23	14.80	0.62	0.539	(-21.31, 39.77)	–
Oxy-Hb change	-111.87***	21.86	-5.12	<0.001	(-156.99, -66.75)	0.785
HAMD score	2.697***	0.57	4.76	<0.001	(1.53, 3.87)	0.769
Gender	1.64	1.88	0.87	0.392	(-2.24, 5.52)	–
Disease duration (months)	-0.13	0.15	-0.91	0.370	(-0.44, 0.17)	–
Suicidal behavior	-1.81	2.04	-0.89	0.383	(-6.01, 2.39)	–
Family history of mental illness	2.08	2.08	1.00	0.327	(-2.21, 6.36)	–
Model : RMT~Oxy-Hb change+HAMD +Covariates	–	–	–	<0.001	–	0.885

***P < 0.001; Model equation: RMT = 9.23 - 111.87×Oxy-Hb change + 2.70×HAMD + 1.64×Gender - 0.13×Disease duration - 1.81×Suicidal behavior + 2.08×Family history.

## Discussion

This study systematically investigated age-related differences in RMT and their underlying neurophysiological mechanisms among patients with depression. Compared with previous research, the present study included subjects with a broader age range, enabling a more robust investigation of the associations between cortical activation and clinical symptoms. We identified distinct age-related patterns in both mean physiological levels and their correlational associations. Regarding mean group differences, the adolescent cohort exhibited significantly higher baseline RMT and the lowest levels of prefrontal Oxy-Hb activation compared to the young adult and middle-aged groups. However, regarding correlational associations, we observed a broadly conserved physiological pattern across all age groups: in all three cohorts, higher RMT was consistently associated with lower cortical activation and more severe depressive symptoms. While this underlying coupling mechanism is present across ages, adolescents represent the most extreme manifestation of this spectrum, with the highest mean RMT, lowest mean cortical activation, and strongest within-group correlations. Moreover, a higher RMT was associated with more severe depressive symptoms in the adolescents group, indicating that this high threshold-low activation-severe symptoms triad represents the most extreme manifestation of a broadly conserved physiological coupling pattern across all age groups. Previous studies have demonstrated that RMT shows a negative correlation with age in all healthy individuals, confirming that age is an independent regulator of RMT unrelated to disease status (25, 26). While declining effective connectivity from the premotor cortex to the primary motor cortex with age, may underlie elevated RMT in healthy aging, this correlation appears to be disrupted under pathological conditions. However, this correlation disappears under pathological conditions such as depression, suggesting that healthy aging and pathological states exert distinct effects on RMT through different mechanisms (27, 28). Age-related differences in RMT among individuals represent a key focus of discussion in this study.

From the perspective of age specificity of RMT, the significantly higher RMT in the adolescents group compared with the young adult and middle-aged groups stands in contrast to the age-dependent decrease in RMT observed in healthy populations. This finding suggests a distinct regulatory mechanism of cortical excitability in depressed adolescents. Although data on RMT in children and adolescent depressed patients remain scarce, existing rTMS studies investigating adult and adolescent depression have emphasized that neurodevelopmental factors (e.g., cortical maturity, alterations in the excitation-inhibition balance of cortical networks) may influence treatment efficacy in adolescents, including RMT-related parameters (29, 36). These findings indirectly indicate that RMT in children and adolescents may differ from that in adults due to distinct developmental stages (30–32). From a neurodevelopmental perspective, several potential neurobiological mechanisms may underlie the observed elevation of RMT in depressed children and adolescents, including abnormal neural circuitry involving the amygdala, hypothalamus, and subgenual anterior cingulate cortex. Current evidence indicates that the maturation of cortical excitability may be delayed in children and adolescents with depression (33, 34). A prior study reported that motor threshold (MT) is markedly elevated in young children with depression and gradually decreases with increasing age (35). From a neurodevelopmental perspective, the adolescent brain is in a critical stage characterized by asynchronous maturation of cortical and subcortical structures: subcortical regions reach a stable state at approximately 10 years of age, and the limbic system (e.g., amygdala, nucleus accumbens)—considered the “accelerator” of emotional responses—approaches maturity in early adolescence, contributing to heightened emotional sensitivity and enhanced reward motivation in adolescents (36, 37). In contrast, the prefrontal cortex (PFC), particularly the DLPFC, which serves as the “brake” system responsible for cognitive control and emotional regulation, undergoes continuous development until early adulthood through myelination and synaptic pruning, with its maturation rate lagging far behind that of subcortical structures. In the context of depression, white matter fiber tracts connecting the PFC and limbic system (e.g., uncinate fasciculus, cingulum bundle) may remain insufficiently myelinated, resulting in inefficient information transmission. This deficiency impairs the top-down inhibitory control of the PFC over the limbic system, leading to a dysregulation of cortical excitability and manifested as elevated RMT. Therefore, the delayed maturation of the advanced regulatory functions of the prefrontal cortex contributes to the exacerbation of behavioral problems and depressive symptoms in adolescents (38–40). Meanwhile, the GABA ergic inhibitory system is still developing during adolescence: the density of GABA receptors decreases with age, and the coordination with the glutamatergic system is insufficient, rendering the cortical excitation-inhibition balance in a highly dynamic and vulnerable state. This may further exacerbate the elevation of RMT and the reduction of cortical excitability, which is consistent with the conclusion of previous studies that “cortical inhibitory function is impaired in depressed adolescents” (41–43).

Regarding the association between RMT and cortical activation, the adolescents group exhibited significantly lower prefrontal Oxy-Hb activation levels compared with the other two groups, and a strong negative correlation was observed between RMT and prefrontal activation (*Spearman’s rs* = -0.886, *P* < 0.001). Although no previous studies have directly examined the relationship between RMT and cortical activation, the “hypofrontality hypothesis” of depression has been proposed, which posits that the hypoexcitable prefrontal cortex in adolescents may be unable to effectively mobilize neural resources during the VFT, resulting in insufficient hemodynamic responses. This hypofrontality is closely associated with cognitive control deficits and emotional regulation disorders caused by cortical excitability inhibition (44, 45). Several neurobiological models may help interpret the observed age−related differences in RMT and prefrontal activation. Previous neuroimaging studies have reported distinct patterns of prefrontal−limbic network connectivity between adolescent and adult patients with depression. For instance, the connectivity between the subgenual anterior cingulate cortex (sgACC) and amygdala remains relatively intact in depressed adolescents but is significantly reduced in adults, especially those with recurrent depression (46). This developmental divergence in emotional circuit architecture may underlie the distinct cortical excitability profiles observed across age groups. From the perspective of neural circuit mechanisms, we hypothesize that the observed hypoexcitable and hypoactive prefrontal cortex might be associated with a failure of top-down regulatory control over limbic structures. Abnormal development of cortico-subcortical functional connectivity during adolescence allows local cortical excitability abnormalities (e.g., elevated RMT) to affect the whole-brain emotional circuit through network effects. The functional connectivity between the prefrontal cortex and limbic system in adolescents is poorer than that in adults, potentially accompanied by a higher degree of imbalanced cortical inhibition, which further amplifies the hypoactivation of the prefrontal cortex. This cycle not only exacerbates the state of insufficient cortical activation but also promotes the progression of depressive symptoms (47, 48). This explains why the correlation between RMT and cortical activation in the adolescents group is significantly stronger than that in the adult groups. However, because our fNIRS and TMS assessments are limited to cortical regions, the specific involvement of the amygdala and deeper prefrontal-limbic networks remains speculative and requires future verification via functional magnetic resonance imaging. In addition, glutamatergic and inflammatory mechanisms have been proposed in adolescent depression. The TIGER study program highlighted that excessive glutamate within corticolimbic circuits may link peripheral inflammation to treatment non−response in adolescents (49).Since the glutamatergic system strongly modulates cortical excitability, developmental differences in glutamatergic regulation likely contribute to the elevated RMT and reduced prefrontal activation seen in our adolescent group. These broader circuit and molecular models provide a theoretical framework for understanding why the high threshold−low activation pattern is most prominent in adolescents, even though they were not directly measured in the current study.

With respect to the associations among RMT, cortical activation, and depression severity, a strong positive correlation was observed between RMT and HAMD scores in the adolescents group (*Spearman’s rs* = 0.878, *P* < 0.01). Additionally, multiple linear regression analysis showed that prefrontal Oxy-Hb changes and HAMD scores remained independent predictors of RMT after controlling for confounding factors such as gender and disease duration. This finding demonstrated that alterations in cerebral oxygenation function may directly modulate cortical excitability in adolescents patients; meanwhile, more severe depressive symptoms were associated with higher cortical excitability in this population. These findings support the specificity and independence of the “high threshold-low activation-severe symptoms” triad. The core mechanism underlying this association lies in the functional chain reaction triggered by the imbalance between the prefrontal cortex and limbic system: hypoactivation of the PFC leads to core depressive symptoms such as cognitive retardation and anhedonia, while the exacerbation of symptoms further impairs the ability of the PFC to mobilize neural resources, forming a negative feedback loop of “symptom-physiological abnormality” (50). Compared with adult depression, the association between depressive symptoms and brain region activation in adolescents appears to be more direct and explicit. In adult depression, complex clinical symptoms cannot be fully explained by the activation level of a single brain region, possibly due to confounding factors such as comorbidities and chronic disease course. In contrast, the neuropathological mechanisms of adolescent depression are more focused on developmentally specific circuit imbalances, enabling RMT and prefrontal activation to serve as objective biomarkers reflecting depression severity (51, 52).

Collectively, our findings underscore substantial divergences between adolescents and adults in neurodevelopmental trajectories and cortico-subcortical coordination. The observed pattern of high RMT combined with low cortical activation in adolescent depression a broadly conserved coupling pattern that is most pronounced in this age group highlights the vulnerability of brain circuits during this critical developmental period. Targeted interventions aimed at this specific circuit may represent a promising avenue for improving the prognosis of adolescent depression. in the future. Current rTMS treatment protocols are mostly adapted from adult regimens, which do not fully consider the neurodevelopmental characteristics of adolescents and may lead to inappropriate stimulation dosages (either excessive or insufficient), thereby affecting treatment efficacy and safety. This study highlights the potential value of TMS threshold in individualized treatment, suggesting that future rTMS therapy for adolescents should adhere to the principle of “developmental stage adaptation”: Target selection: Priority should be given to key prefrontal regions such as the DLPFC to promote the maturation of functional connectivity with the limbic system; dosage setting: The significantly elevated baseline RMT in adolescents presents distinct clinical challenges. Empirically, applying standard therapeutic ratios (e.g., 120% RMT) to a high baseline requires absolute stimulator outputs that frequently cause severe scalp pain and facial twitching, thereby reducing treatment tolerability and adherence in younger patients. Speculatively, administering higher absolute magnetic field intensities to a developing brain might theoretically influence the risk profile for adverse events such as seizures, warranting cautious parameter titration. Parameter optimization: fNIRS can be used to monitor prefrontal activation levels, enabling dynamic optimization of treatment parameters to ensure that interventions effectively ameliorate the “high threshold-low activation” state and promote the healthy integration of emotional circuits.

It should be noted that this study has several limitations. First, the cross-sectional design precludes inference regarding causal relationships. Additionally, because this study focused exclusively on baseline neurophysiological evaluations, longitudinal data regarding the patients’ subsequent clinical responses to the full rTMS treatment course were not available, precluding exploratory analyses linking baseline RMT or fNIRS metrics to ultimate therapeutic outcomes. Second, while the *post-hoc* power analysis confirmed that the sample size was statistically adequate to detect the large effects observed in the current primary analyses, the overall cohort size remains relatively small, which may limit the broader generalizability of the findings. Third, the absence of a healthy control group prevents the exclusion of the influence of normal developmental variations. Fourth, not all confounding factors (e.g., the precise effects of antidepressant medications) were fully controlled. Fifth, the limited spatial resolution of fNIRS may have overlooked changes in deep brain regions. Sixth, the fNIRS topographic maps were generated via automated clinical software primarily for descriptive visualization. Due to system constraints, strict channel-wise statistical thresholding (such as False Discovery Rate correction) was not applied, restricting our ability to perform highly localized, region-specific functional mapping. Future studies should employ longitudinal designs, expand the sample size, and combine multimodal imaging techniques (e.g., functional magnetic resonance imaging, fMRI) to further verify these conclusions. Nevertheless, overall, this study clarifies the neurophysiological characteristics of the “high threshold-low activation-severe symptoms” triad in adolescent depression and its development-related mechanisms, providing important theoretical support for the objective assessment of depression severity and precise neuromodulation in this population.

## Conclusion

This study identified a broadly conserved high resting motor threshold - low cortical activation - severe depressive symptoms coupling pattern that is most pronounced in adolescents through cross-age differential analysis. This finding provides critical theoretical support for the precise assessment and targeted neuromodulation in adolescents with depression, indicating that interventions for adolescent depression should prioritize promoting the healthy integration of emotional circuits and the functional coordination between the cortex and subcortex.

## Data Availability

The original contributions presented in the study are included in the article/supplementary material. Further inquiries can be directed to the corresponding authors.
